# Targeting PI3K-mTOR signaling in the anterior cingulate cortex improves emotional behavior, and locomotor activity in rats with bone cancer pain

**DOI:** 10.1097/MS9.0000000000003206

**Published:** 2025-03-19

**Authors:** Shuyun Liu, Rujia Zhu, Yuan Zhang, Zongming Jiang, Yonghao Chen, Qiliang Song, Fei Wang

**Affiliations:** a Department of Anesthesiology, Shaoxing People’s Hospital, Shaoxing, China; b School of Medicine, Shaoxing University, Shaoxing, China; c Department of Anesthesiology, Shanghai Jiang Qiao Hospital, Shanghai, China; d Bioinformation Branch, Hangzhou Hibio Bioinformation Technology Company, HangZhou, China

**Keywords:** anterior cingulate cortex, bone cancer pain, emotion regulation, locomotion, phosphoinositide 3 kinase

## Abstract

**Objective:**

To investigate the effects of targeting the PI3K-mTOR signaling pathway in the anterior cingulate cortex (ACC) on pain responses, locomotor activity, and emotional behavior in rats with bone cancer pain.

**Methods:**

Bone cancer pain was induced by implanting Walker 256 cells into the rat. Pain responses were assessed using paw withdrawal threshold and latency measurements, while locomotor activity and negative mood were evaluated through open field and conditioned place aversion tests, respectively.

**Results:**

The results showed that the bone cancer pain model led to allodynia, hyperalgesia, decreased ambulation, and ACC microglial activation. Morphine treatment improved pain responses but did not affect locomotor activity or mTOR protein expression. In contrast, rapamycin treatment reduced pain, improved locomotor activity, and decreased negative mood. It also downregulated PI3K-mTOR protein expression. Furthermore, inhibiting the PI3K-mTOR pathway with a PI3K inhibitor or rapamycin not only improved pain responses and locomotor activity but also reduced depression and anxiety-like behaviors. These effects were accompanied by changes in paw withdrawal threshold, latency, static time, and PI3K-mTOR protein expression.

**Conclusions:**

Targeting the PI3K-mTOR signaling pathway in the ACC effectively alleviates pain-related symptoms and emotional disturbances in rats with bone cancer pain. This approach holds promise for alleviating pain and allaying negative emotion after further study.

## Introduction

Pain is a complex event consisting of sensory and affective components. The increasing prevalence of cancer-related pain corresponds to the rising incidence of cancer^[1]^. This pain primarily results from tumor growth, inflammation, viral infection, and nerve injury, as tumors compress or infiltrate adjacent tissues or induce neuroinflammation in central body regions. Cancer-related pain is often debilitating or intractable and leads to emotional and psychological changes (affective pain), such as reduced social activities, anxiety, and depression^[1,2]^. These emotional components impact the quality of life more significantly than the pain itself and often form a vicious cycle with pain, exacerbating each other. Negative emotions, including severe pain, anxiety, fear, and depression, are widely recognized to affect the quality of life^[2,3]^. Although numerous analgesics have been used in clinical practice to counteract pain, complete and effective pain relief remains elusive. This is partly due to the limited understanding of how negative emotions contribute to pain.

**HIGHLIGHTS**

- The emotional component of pain impacts the quality of life more significantly than the pain itself.
- The anterior cingulate cortex plays a crucial role in encoding pain, especially emotional pain.
- Targeting the PI3K-mTOR signaling pathway in the ACC effectively alleviates pain-related symptoms and emotional disturbances.
- It holds promise for improving pain management and emotional well-being.

The mammalian target of rapamycin (mTOR) consists of two distinct protein complexes: mTOR complex 1 (mTORC1) and mTORC2. The regulation of the mTOR pathway significantly impacts cell growth, proliferation, survival, and hormone regulation^[4]^. Specifically, mTORC1 induction controls gene expression at the translational level by phosphorylating downstream effectors, including 4E-binding proteins (4E-BPs, eukaryotic initiation factors) and p70 ribosomal S6 protein kinases (70S6Ks). In mammalians, mTOR, 70S6K1, and 4E-BP1 are produced by the nervous system, particularly in the spinal cord dorsal horn and cerebral areas^[5]^, where they control the relay and regulation of nociceptive signals^[6]^. The interaction between cells is realized through signal transduction. PI3K exist in cytoplasm and has dual activity of protein kinase and phospholipkinase. PI3K is activated by cytokines and growth factors and acts on Akt protein to activate or inhibit a series of downstream substrates, among which mTOR is the downstream target of PI3K/Akt^[7]^. PI3K-mTOR signaling pathway are signal transduction pathways involved in the regulation of cellular functions, including cell proliferation, survival, differentiation, adhesion, motility, and invasion^[8]^.

Rapamycin is a first-generation mTOR inhibitor^[7]^. A previous study using Walker 256 cells to establish a bone cancer model found that administering rapamycin to the spinal cord suppressed mTOR, significantly alleviating pain and simultaneously ameliorating the severity of morphine tolerance in rats^[8]^. The potential molecular mechanism involves the participation of opioid µ-receptors and the induction of mTOR and downstream effectors (70S6K1, 4E-BP1, etc.), which contribute to pain modulation. Another study reported a significant decrease in locomotor activities and pain intensity in rats following rapamycin infusion to the spinal cord^[9]^. However, these studies were concentrated on the relationships between mTOR in the spinal cord and pain behaviors, particularly the data about the activation of mTOR at brain level and emotional change were limited. Hence, we hypothesized that, in addition to the spinal cord, the emotional response and locomotor activity of rats are regulated at higher level and are closely associated with mTOR family molecules. The precise delineation of this will be helpful for deciphering the role of mTOR in modulating pain related emotion fluctuation and provide new potential therapeutic consideration.

The anterior cingulate cortex (ACC) plays a crucial role in processing pain, especially emotional pain, and responds to noxious and contextual stimuli^[10-12]^. The ACC is involved in modulating cognition and pain-related emotions, including empathy, aversion, and unpleasantness^[13]^. Additionally, ACC neurons facilitate the transmission of pain signals by regulating spinal cord neurons^[14]^. Despite opioids being the primary drugs used for pain management, their chronic administration can lead to physical dependence and affective changes, complicating pain treatment. Given the ACC’s role in pain processing and the limitations of opioid treatments, we hypothesized that mTOR in the ACC may contribute to the development of morphine tolerance and negative emotions.

In the present study, we aimed to investigate the role of PI3K-mTOR signaling in the ACC on pain responses, locomotor activity, and emotional behavior in rats with bone cancer pain. We speculated that the findings will highlight the importance of affective pain in the regulation of pain-related behaviors and provide new insights for pain related emotion change.

## Materials and methods

### Animals

Male Wistar rats (200–250 g) were obtained from the Institutional Center of Experimental Animals (Shanghai SLAC Laboratory Animal, China) and housed at 22 ± 2 °C under a 12-hour light-dark cycle, with access to rodent chow and water ad libitum. The animal protocols were approved by the Animal Care and Use Committee of Shaoxing People’s Hospital, Shaoxing, China (No: 2020-79) and followed the guidelines of the International Association for the Study of Pain. The current work we displayed here in alignment with the ARRIVE criteria^[15]^. Rats were allowed to acclimate to their housing environment for 4–5 days before each intervention.

### Bone cancer pain model

To establish the bone cancer pain model, rats were randomly divided into sham and model groups (n = 8–10 per group).In our pretesting study, we found that some rats died prior to the completion of experiment due to the filtration of tumor or other reasons. After the power analysis, the total number of 96–100 rats was needed to identify the difference. An independent assistant experimentalist known to group allocation conducted the measurements and evaluations. Bone cancer pain was induced in the model group by injecting human breast sarcoma Walker 256 cells into the left tibia, as previously described^[15,16]^. Specifically, the rats were lying on their back after anesthetized by intraperitoneal injection of 50 mg/kg sodium pentobarbital, the left hind limb was clipped and disinfected with 75% ethanol. A small concave was drilled into the tibia with a 10 ml syringe needle, and then a dental drill was used to penetrate the bone cortex into the bone marrow cavity. 5 μL of Hank’s solution containing 1 × 10^5^ resuspended cells was administered to the medullary cavity. Seal the hole with bone wax and saline was used to clean the wound and then sutured the skin with wound clips. In the sham group, rats underwent tibial osteotomy drilling without cancer cell injection. Applying erythromycin eye ointment to the incisional site to prevent infection for all rats. At the end of each intervention, butorphanol or ketamine was intraperitoneally administered to alleviate pain. Wound clips were discarded 5–6 days after surgery under sevoflurane anesthesia.

### Intracerebral microinjection and sample collection

After the successful establishment of the rat cancer pain model, the head of the anesthetized rat was fixed on the stereoscope to ensure that the head was stable under sevoflurane inhalation anesthesia(#2300451, Jiangsu Hengrui Medicine, China). After the location of the ACC was determined by the stereoscope, drilled a hole in the exact location, and then the stainless steel guide Pin (24 G) was slowly implanted^[16,17]^. Each cannula was fixed to the skull with anchoring screws and sterile acrylic cement. Simultaneously, applying erythromycin eye ointment to the incisional site to prevent infection. To prevent occlusion, a dummy 28 G cannula was inserted and removed two to three times per day. Timepoints and dosage for microinjection were based on previous studies^[8,10,13]^.

Micro-infusion was performed for 3 minutes, with the injection cannula retained for 5 minutes after drug infusion to minimize drug reflux. Drugs, including rapamycin (10 ng/0.5 μL/side; Selleckchem, China), morphine (1 μg/0.5 μL/side; Humanwell, China), morphine plus rapamycin (1 μg morphine plus 10 ng rapamycin in total, 0.5 μL/side), PI3K inhibitor (LY294002, 5 μg/0.5 μL/side; Sigma, USA), and vehicle (1% dimethyl sulfoxide, 0.5 μL/side; ThermoFisher Scientific, USA), were administered via an osmotic micropump (Model 2004/2006, Alzet Osmotic Pumps, DURECT, USA). The drugs were dissolved in 50% dimethyl sulfoxide to generate stock solutions, which were then diluted with saline to achieve the target levels at the time of infusion. After completion of all experiments, site verification was performed by administering 0.25 μL of 2% Evans blue through the cannula. The animals were then decapitated, and brain tissues in the ACC were collected for further research.

### Measurement of PWT and PWL

Mechanical allodynia was assessed by evaluating the paw withdrawal threshold (PWT) following stimulation with calibrated von Frey hairs (Stoelting Yuyan Instruments, China). Animals were placed on a 30-cm high metal wire and allowed to acclimate for 30 minutes. Von Frey filaments (1.0, 2.5, 3.5, 4.0, 4.5, 5.0, 7.5, 10.0, 20.0, 30.0, 40.0, 50.0, and 60.0 g) were used to stimulate the plantar surface of the hind paw, with intensity increasing sequentially. Starting with the 1.0-g filament, the test progressed upward in the absence of a response (= 0) or downward upon sudden limb withdrawal (= X), until all evaluations were conducted near the threshold point. Each intensity was applied 10 times with a 3–5 seconds interval or until paw withdrawal (hind paw completely removed from the platform). The minimal intensity eliciting a ≥ 50% response was considered the PWT and calculated as follows:

$$50\%\;g\;Threshold=\frac{\left(10^{Xf+k.\partial }\right)}{10000}$$

Here, Xf represents the value (log units) of the final von Frey monofilament, and k signifies the tubular value reflecting the pattern of positive and negative responses (X and 0 sequence) between stimuli.

An RTY3 thermal stimulator was used to irradiate the mid-left hind limb, with the heat source turned off when the animal lifted its foot. Measurements were taken three times at 10-minute intervals, averaged, and recorded as the paw withdrawal latency (PWL). To prevent tissue damage, an automatic cut-off latency of 30 seconds was implemented during measurements. PWT and PWL were measured on day 0 (before modeling) and days 1, 4, 7, and 14 after modeling.

### Open field test

To assess spontaneous and active locomotor activities under various interventions, animals were tested using an open field apparatus (JLBehv-LAG-4, Shanghai Jiliang Software Technology). Each animal was placed in the center of the apparatus, allowing the test rat to freely explore the area. The apparatus featured an integrated video monitoring system that captured the movements of the rats and automatically analyzed the images. After each test, the open field chamber was cleaned with 75% ethyl alcohol. The soundproof apparatus, maintained at an ambient temperature of 24 ± 2 °C, provided soft background lighting. Testing occurred between 8:30 am and 11:30 am, with efforts made to prevent unintentional disturbance that could affect the results.

### Conditioned place aversion

Using a conditioned place avoidance (CPA) paradigm can cause significant aversion to a location associated with pain-related insults in rats. The conditional avoidance apparatus was composed of three white polyvinyl chloride plastic chambers: two large conditioning compartments (30 cm × 30 cm × 30 cm) and one small neutral compartment (15 cm × 10 cm × 30 cm). To create distinct visual, tactile, and olfactory stimuli in the two large compartments^[18]^, one featured horizontal strips with 1% acetic acid, while the other had vertical strips and different floor textures (one smooth and one with straight soft padding). The middle room had a neutral wall color, with no specific odor. A removable plastic door was installed between the three compartments for isolation.

**Figure 1.**
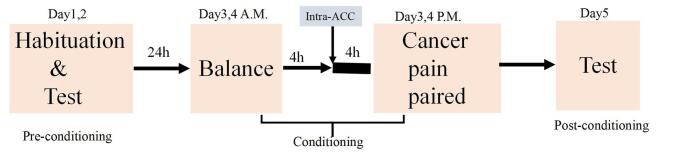
Test procedure for conditioned place aversion (CPA).

Each measurement was conducted between 6:00 am and 6:00 pm without light stimulation and included three different sessions: preconditioning on days 1 and 2, conditioning on days 3 and 4, and postconditioning on day 5. On day 1, rats were placed in the middle compartment for 5 minutes, and the doors to the conditioning compartments were opened, allowing the rats to freely explore for 15 minutes. The analysis software recorded and analyzed the paths taken and time spent in each compartment. This test was repeated on day 2. Rats that spent over 720 seconds in a single ‎compartment on day 2, or over 600 seconds on day 1, were excluded from subsequent analyses. On days 3 and 4, the conditional tests were conducted with the compartment doors closed, and the rats were randomly placed in one of the conditioning compartments for 45 minutes to assess their preference for entering that compartment. The test procedure is illustrated in Fig. 1. Open field test and CPA were assessed on days 7 and 14 to characterize the pain model.

### Immunohistochemistry

ACC tissue samples were collected and sectioned into 4-µm slices. These slices underwent xylene dewaxing, rehydration through gradient alcohol series, a 15-minute incubation with 3% H_2_O_2_ at 37 °C, three washes, and a 10-minute incubation in 0.01 M oleic acid buffer (pH = 6.0). After cooling to room temperature, the slices were incubated overnight with anti-OX-42 primary antibody (1:100, mouse polyclonal IgG; Abcam, UK) at 4 °C. Finally, a 30-minute incubation with secondary antibodies (Abcam) was carried out at 37 °C, followed by DAB staining and hematoxylin counterstaining. Neuron imaging was conducted using an Olympus BX43 fluorescence microscope. On day 14, rats were euthanized to determine OX-42 expression in the ACC.

### Western blotting

The animals were euthanized at various time points following drug infusion, and brain tissue was quickly dissected and collected. Anatomical microscopy-guided dissection was used to extract ACC tissues on ice. Then, 0.1 mg of tissue was placed in an Eppendorf tube containing 500 μL lysis buffer, thoroughly homogenized and centrifuged at 4 °C and 11 000 rpm for 20 minutes. The resulting supernatants were stored at −80 °C. Protein concentrations were determined using the BCA kit. Equal amounts of total protein (20 μg) were separated by SDS-PAGE and subsequently transferred onto a polyvinylidene fluoride membrane. The membrane was then incubated with 5% BSA at room temperature for 1 hour. Next, the membrane was incubated overnight with the following primary antibodies at 4 °C: rabbit anti-p-mTOR (1:500, ab8440, Cell Signaling, USA), rabbit anti-p-P70s6k (1:500, ab60948, Cell Signaling), mouse anti-p-PI3K (1:500, ab182651, GENETEX), rabbit anti-p-4E-BP (1:500, ab2606, Cell Signaling), and rabbit anti-β-tubulin (1:500, CB65591556, Multi Sciences, China). Following the primary antibody incubation, goat anti-rabbit and anti-mouse IgG (1:5000, Cell Signaling) were added as secondary antibodies, respectively, and incubated at room temperature for 2 hours. Lastly, equal parts of solutions A and B from the ECL kit were applied to the membrane’s surface, which was then transferred to a gel imaging analyzer for chemiluminescent detection. The optical density of protein bands was quantified using the NIH Scion image Software.

### Statistical analysis

Initially, for animal numbers, we did not predetermine the sample size but based on previous trial^[8]^. Then, the sample sizes were calculated using GPower 3.1 software to guarantee sufficient statistical difference for each group to detect 0.8 of two tailed at *P* level below 0.05. Data analysis was performed using GraphPad Prism 7.0 (GraphPad Inc, San Diego, CA) or SPSS Statistics (IBM Inc, Chicago, IL). All data were tested using the Shaprio–Wilk normal distribution test prior to analyze. Normally distributed parameters were presented as mean ± standard deviation. For comparisons between paired or unpaired groups, the Student’s t-test was employed. Multiple group comparisons were conducted using one-way or two-way ANOVA followed by a post hoc Bonferroni test. A *P* value of less than 0.05 was deemed to be statistically significant.

## Results

### Characterization of bone cancer pain model

**Figure 2.**
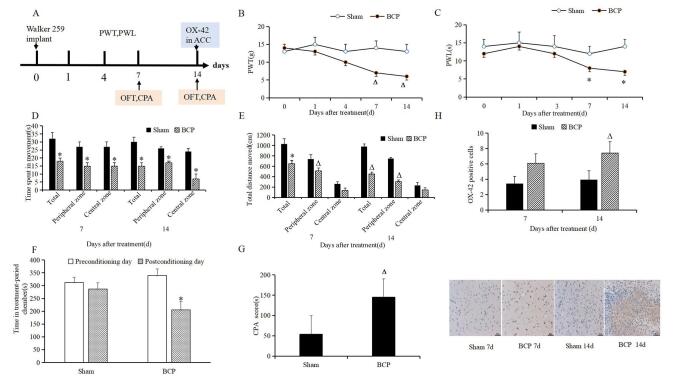
Characterization of the bone cancer pain model. (A) Rats were randomly divided into sham and model groups. Bone cancer pain was induced in the model group by injecting human breast sarcoma Walker 256 cells into the right tibia. Open field test and CPA were assessed on day 7 and 14. (B, C) Paw withdrawal threshold (PWT) and latency (PWL) were measured on day 0 (before modeling) and on days 1, 4, 7, and 14 after modeling. (D, E) Time spent moving and total distance travelled in the open field test. (F, G) Time spent in treatment-paired chambers and CPA scores in the CPA assay. (H) On day 14, rats were euthanized. Immunohistochemistry was performed to detect OX-42 expression in the anterior cingulate cortex (ACC). Scale bar = 50 µm. Data are expressed as mean ± standard deviation (SD). ACC: anterior cingulate cortex; PWT: pain withdrawal threshold; PWL: pain withdrawal latency; CPA: conditioned place aversion; BCP: bone cancer pain. ^Δ^*P* < 0.05, *P < 0.01, ^#^*P* < 0.001 vs. the sham group; n = 6.

To confirm the successful establishment of the bone cancer pain model, we performed open field tests and CPA assays at various time points before and after modeling (Fig. 2A). On day 7 after implanting Walker 256 cells into the rat tibia, mechanical allodynia and thermal hyperalgesia were observed and continued to develop by day 14. This was demonstrated by the significant decrease in PWT and PWL in the bone cancer pain model rats on both the 7th and 14th days (*P <* 0.05; Fig. 2B and C). In the open field test, the pain group showed a significant decrease in time spent moving and total distance traveled compared to sham rats (*P <* 0.05, *P* < 0.01; Fig. 2D and 2E). In the CPA assay, pain rats spent less time in treatment-paired chambers and exhibited higher CPA scores (*P <* 0.01, *P* < 0.001; Fig. 2F and 2G).

To investigate the impact of bone cancer pain on ACC histology, we performed immunohistochemistry. The results showed a significant increase in OX-42-expressing cells, a marker for activated microglial cells at the ACC site, in the pain group on days 7 and 14 compared to sham rats (*P <* 0.05, *P* < 0.01; Fig. 2H). We established a link between bone cancer pain and changes in the ACC, based on behavioral performance and ACC glial cell activation. As a result, we continued our study using rats with Walker 256 cell implantation for 14 days.

***Morphine alleviates pain but fails to improve movement and mTOR-related protein expression in the bone cancer pain model rats***

**Figure 3.**
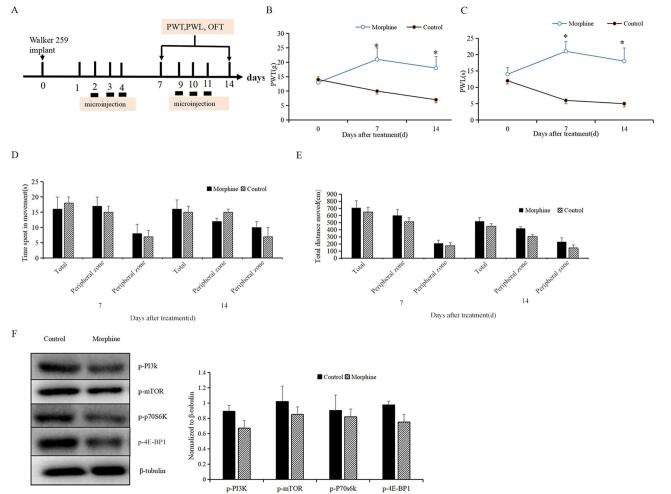
Effects of microinjection of morphine into the ACC on pain sensitivity and locomotor activity. (A) Bone cancer pain model rats were divided into two groups and received either morphine (1 μg/0.5 μL/side) or vehicle (1% DMSO, 0.5 μL/side) injections into the ACC via an osmotic micropump on days 2, 3, 4, 9, 10, and 11 after modeling. (B, C) PWT and PWL were measured on days 7 and 14. (D, E) Time spent moving and total distance travelled in the open field test on days 7 and 14. (F) On day 28, rats were euthanized. Western blot analysis was performed to determine protein levels of p-PI3K, p-mTOR, p-4E-BP1, and p-70s6k in the ACC. Data are expressed as mean ± SD. ACC: anterior cingulate cortex. PWT: pain withdrawal threshold; PWL: pain withdrawal latency: CPA: conditioned place aversion. ^Δ^*P* < 0.05, *P < 0.01, ^#^*P* < 0.001 vs. vehicle; n = 5.

To evaluate the efficacy of morphine for bone cancer pain relief, we treated the pain model rats with vehicle or morphine on days 2, 3, 4, 9, 10, and 11 after modeling (Fig. 3A). The micro-infusion of morphine into the ACC of model rats significantly increased both PWT and PWL (*P <* 0.05, *P* < 0.01; Fig. 3B and 3C). However, there were no noticeable differences in total distance and time spent moving on days 7 and 14 between the two groups (Fig. 3D and 3E). Moreover, on the 28th day, the morphine group did not exhibit any advantageous alterations in the levels of p-PI3K, p-mTOR, p-4E-BP1, and p-70s6k when compared to the vehicle group (Fig. 3F). These results suggest that while morphine infusion substantially decreased pain intensity and sensitivity, it did not enhance the rats’ movement status or reduce the expression of mTOR-related proteins.

### Rapamycin alleviates bone cancer pain and improves emotional behavior in rats

**Figure 4.**
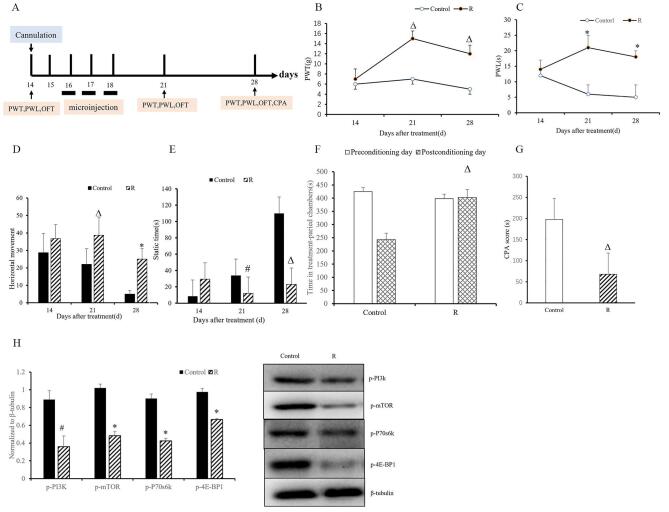
Administration of rapamycin to the ACC attenuated pain sensitivity and ameliorated pain-related aversion in the bone cancer pain model. (A) Bone cancer pain model rats were divided into two groups and received either rapamycin (10 ng/0.5 μL/side) or vehicle (1% DMSO, 0.5 μL/side) injections into the ACC via an osmotic micropump on days 16, 17, and 18 after modeling. (B, C) PWT and PWL were measured before treatment (day 14) and after treatment (days 21 and 28). (D, E) Horizontal and static time in the open field test on days 21 and 28. (F, G) Time spent in treatment-paired chambers and CPA scores in the CPA assay on day 28. (H) On day 28, rats were euthanized. Western blot analysis was performed to determine protein levels of p-PI3K, p-mTOR, p-4E-BP1, and p-70s6k in the ACC. Data are expressed as mean ± SD. ACC: anterior cingulate cortex; PWT: pain withdrawal threshold; PWL: pain withdrawal latency; CPA: conditioned place aversion. ^Δ^*P* < 0.05, *P < 0.01, ^#^*P* < 0.001 vs. vehicle; n = 7.

To investigate the involvement of mTOR signaling in bone cancer pain, we treated the model rats with rapamycin or vehicle on days 16, 17, and 18 after modeling (Fig. 4A). Rapamycin treatment reduced pain intensity, as evidenced by higher PWT and PWL compared to vehicle treatment (*P <* 0.001; Fig. 4B and 4C). Consistent with these findings, the open field test showed a significant increase in horizontal movement and a notable decrease in static time in rapamycin-treated rats compared to vehicle-treated rats on days 21 and 28 (*P <* 0.05; Fig. 4D and 4E). To assess the effect of rapamycin on emotional behavior, we compared CPA scores before and after rapamycin administration. We observed increased time spent in treatment-associated chambers and lower CPA scores (*P <* 0.01, *P* < 0.001; Fig. 4F and 4G). Rapamycin administration significantly reduced the ‎levels of p-‎PI3K, p-mTOR, p-E-BP1, and p-70s6k on day 28 compared to the vehicle treatment (*P <* 0.01, Fig. 4H). These results suggest that rapamycin not only alleviates bone cancer pain intensity but also helps to mitigate the negative mood caused by pain by modulating PI3K/mTOR signaling pathway.

***Inhibition of PI3K-mTOR signaling pathway modulates pain response and behavioral changes in rats with ACC-targeted drug administration***

**Figure 5.**
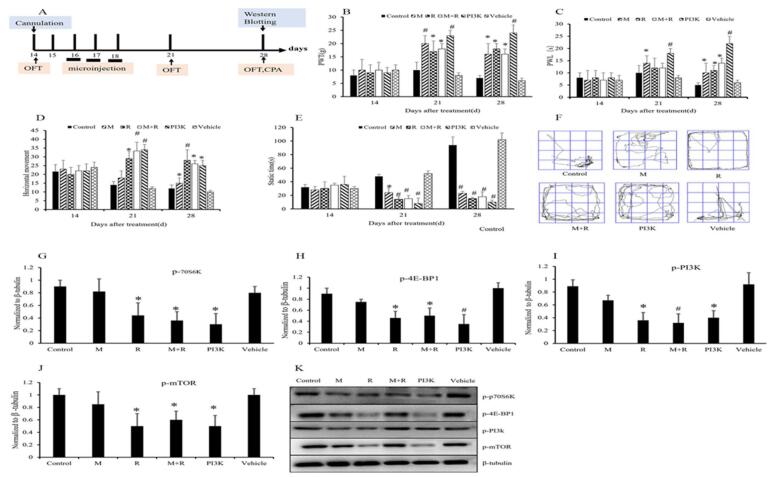
Modulation of pain response and behavioral changes in rats with ACC-targeted drug administration through inhibition of the PI3K-mTOR signaling pathway. (A) Bone cancer pain model rats were divided into six groups and received vehicle (1% DMSO, 0.5 μL/side), rapamycin (10 ng/0.5 μL/side), morphine (1 μg/0.5 μL/side), morphine plus rapamycin (1 μg morphine plus 10 ng rapamycin in total, 0.5 μL/side), or PI3K inhibitor (LY294002, 5 μg/0.5 μL/side) injections into the ACC via an osmotic micropump on days 16, 17, and 18 after modeling. (B, C) PWT and PWL were measured before treatment (day 14) and after treatment (days 21 and 28). (D, E) Horizontal and static time in the open field test on days 21 and 28. (F) The representative paths of the rats in open field test. On day 28, rats were euthanized. (G, H, I, J) Western blot analysis was performed to determine protein levels of p-‎PI3K, p-mTOR, p-E-BP1, and p-70s6k in the ACC after microinjection of morphine, rapamycin, morphine combined rapamycin, PI3K inhibitor and vehicle (DMSO) respectively. (K) Typical bands for Western blotting analysis. ACC: anterior cingulate cortex; PWT: pain withdrawal threshold; PWL: pain withdrawal latency; CPA: conditioned place aversion; DMSO: dimethyl sulfoxide. Data are expressed as mean ± SD. *P < 0.01, ^#^ *P* < 0.001 vs. the corresponding group; n = 6.

To investigate whether inhibiting the PI3K-mTOR signaling pathway in the ACC could influence pain response and behavioral changes, we compared the effects of administering morphine, rapamycin, the combination of morphine and rapamycin, and a PI3K inhibitor on the model rats (Fig. 5A). When drugs were administered to the ACC, both PWT and PWL increased in the morphine, rapamycin, morphine + rapamycin, and PI3K inhibitor groups on days 21 and 28 compared to the vehicle-treated group (Fig. 5B and 5C). PWT and PWL were comparable between the morphine and rapamycin groups at corresponding time points, despite increased horizontal movement and decreased static time in the latter. The combination of morphine and rapamycin was comparable to rapamycin alone in pain and locomotor ability (Fig. 5B–E). Morphine and rapamycin did not provide additional pain or improve locomotor ability. There were markedly reduced hypersensitivity and allodynia, improved locomotor ability, and decreased static time on days 21 and 28 in the PI3K inhibitor group, suggesting a decrease in depression and anxiety severity. Improved locomotor ability and anxiety levels were observed in rapamycin and morphine + rapamycin groups compared to morphine group (Fig. 5D and 5E). The PI3K inhibitor group did not show increased movement ability compared to rapamycin or morphine + rapamycin group, despite a significant decrease in static time (Fig. 5E). Representative paths of the rats in the open field test are shown in Fig. 5F.

The expression of p-mTOR, p-p70s6k, and p-4E-BP1 proteins, crucial components of the PI3K signaling pathway, was downregulated in the rapamycin, morphine + rapamycin, and PI3K inhibitor groups. This indicates their involvement in pain responses and behavioral changes, especially emotionality. Compared to control values, the levels of p-mTOR, p-p70s6k, and p-4E-BP1 were significantly reduced (*P <* 0.01, *P* < 0.001; Fig. 5G–K). The levels of p-mTOR, p-p70s6k, and p-4E-BP1 were similar among rapamycin, morphine + rapamycin, and the PI3K inhibitor groups. These results suggest that inhibiting the PI3K-mTOR signaling pathway in the ACC can modulate pain response and behavioral changes, potentially improving locomotor ability and reducing depression and anxiety severity in rats experiencing pain.

## Discussion

This study demonstrated that cancer pain affects pain sensitivity, allodynia, and movement while activating glial OX-42 positive cells in the ACC. It showed that morphine administration reduced pain intensity, while PI3K-mTOR pathway inhibition offered broader benefits, such as pain relief and improved movement. These findings emphasize the ACC’s role in processing pain-related behavioral changes and negative emotions and suggest the potential of PI3K-mTOR pathway inhibition in improving pain management and emotional well-being for cancer pain patients.

Pain encompasses both sensory and affective dimensions, with different nerve conduction pathways and brain regions responsible for processing these components^[19]^. The ACC is closely related to pain-related functions, particularly unpleasantness and aversion, as confirmed through clinical observations, electrophysiological experiments, human brain function imaging, and animal ACC resection experiments^[13,20,21]^. Pain-associated emotional information is mainly encoded in the ACC^[22]^. Horizontal, static time, movement time, and total distance in open field tests are often used as indices of anxiety and locomotion activity^[23]^. Decreased horizontal and total distance indicates higher anxiety levels, while increased static time reflects reduced locomotion. In this study, total distance travelled and time spent in movement significantly decreased after cancer cell implantation, suggesting that pain impacts locomotor activity and active movement range. Persistent pain can produce unpleasantness, depressive mood, and negative emotions^[24]^, as evidenced by high CPA scores observed in rats with bone cancer pain in the present study.

µ-Opioid receptors are widely distributed in brain regions like the thalamus, ventricle, and periaqueductal gray matter, which contribute to pain integration and perception^[25]^. The ACC contains numerous µ-opioid receptors that relieve pain and are involved in conditional preference^[26]^. Morphine, an opioid receptor agonist, produces analgesic effects by activating µ-opioid receptors, improving anxiety, tension, fear, and other pain-related emotional responses while also inducing sedation and increasing pain tolerance^[23]^. In our study with rats experiencing bone cancer pain, morphine microinjection into the ACC increased pain thresholds but did not significantly affect locomotor activity or CPA, which represent negative emotions caused by pain. This highlights the role of µ-opioid receptors in the ACC in modulating mechanical allodynia and hyperalgesia, although pain alleviation did not prominently impact movement and CPA. This disparity may be due to different afferent pathways controlling pain sensory resolution and pain-related emotions^[27]^. Furthermore, longer static time, which reflects anxiety and depression levels, often leads to poor locomotion ability^[23]^.

PI3K-mTOR signaling plays a crucial role in cell proliferation and apoptosis, with PI3K acting as the initial hub this pathway^[28]^, which regulates the cell cycle, tumor cell proliferation, migration, invasion, and angiogenesis^[29,30]^. Recently evidence suggests that PI3K-mTOR signaling is involved in cancer, inflammation, and neuropathic pain management, particularly through mTOR activation^[31-33]^. However, past studies primarily focused on spinal or central mechanisms for pain management, not on behavioral responses and emotional changes. In the present study, we used rapamycin to block mTOR and LY294002 to inhibit PI3K. Both treatments improved spontaneous locomotor activity and ameliorated CPA, indicating PI3K-mTOR signaling regulates activity and emotion (Fig. 5). In line with these behavioral changes, the protein levels of p-PI3K, p-mTOR, and mTOR-regulated downstream molecules (p-4EBP1, p-S6K1) decreased, suggesting that PI3K-mTOR signaling effectors contribute to cancer pain development, supporting previous findings that mTOR activation is crucial for pain behavior expression^[34]^.

Intriguingly, in our current study, contrary to our common intuition that the combination of morphine and rapamycin did not confer better synergic effects (pain and emotional response) than them alone. There were several reasons for the results: (1) morphine acts on µ-opioid receptors and activates NMDA receptors, causing activation of the PI3K-Akt-mTOR signaling pathway. It is an important way for the development of morphine tolerance and hyperalgesia^[35]^ and microinjection of NMDAR antagonists into the ACC to block excitatory synaptic transmission significantly in chronic pancreatitis rats. Obviously, the administration of morphine to ACC exerted analgesic effect and alleviated pain intensity. (2) In spinal cord level, spinal Rheb gates mTOR in regulating pain tolerance^[36]^. In this bone cancer pain model, we could not define how and what extent of Rheb/mTOR signaling in modulating pain and emotion. (3) There was evidence demonstrated that opposing effects on nociception were observed when activation of μ and κ opioid receptor in the ACC^[37]^. Besides μ opioid receptor, morphine also activate κ opioid receptor in the ACC^[38]^, whether this effect affects the emotional change in an animal with existing pain anxiety remains unknown. (4) Cancer pain stirs neuroinflammation, upregulates the expression of mTOR in the ACC, whether morphine and rapamycin produced conflicting effects on emotional change needs to be clarified^[39,40]^. Therefore, the phenomena we observed in this research were not the sole determinant but the complicated interactions between multiple factors. In the future, more studies are necessary for a clear delineation.

Using formalin-induced CPA or the place escape/avoidance paradigm, multiple animal behavioral studies have established a potential regulatory function of the ACC in emotion-like reactions^[20,41]^. Moreover, clinical brain imaging tests have revealed a strong correlation between somatosensory cortical neuron activity and noxious stimulus intensity, while the ACC is linked to subjective discomfort^[22,42]^. In this study, inhibiting PI3K-mTOR signaling led to increased exploration and decreased CPA scores. This could be because PI3K-mTOR signaling influences both central and peripheral pain sensitization. The complex relationship between emotions and exploratory activity makes it difficult to determine if reduced pain levels result in increased movement or if changes in emotions (such as depression or anxiety) improve exploratory behavior. Additionally, previous research has established clear connections between the ACC and anxiety-depressive outcomes in a mouse model^[43]^, further supporting the idea that the ACC plays a critical role in pain-related depression and that PI3K-mTOR signaling may be essential in modulating the affective aspect. Whereas, other signaling like BDNF/TrKB/CREB or neural circuits will participate in the modulation of affective pain^[44,45]^. Unfortunately, we concentrated on PI3K-mTOR signaling in the present study.

In addition to examining emotional shifts due to cancer pain, we also determined OX-42 expression in microglial cells within the ACC. Upon inhibition of the PI3K-mTOR signaling pathway using PI3K and mTOR inhibitors, OX-42 expression was downregulated. It is well-established that OX-42 serves as a reliable indicator of microglia status^[45]^, with high expression typically signifying excessive microglia activation. Our findings indicate that microglia were activated in the ACC of rats with bone cancer pain, and PI3K-mTOR inhibitors successfully dampened this activation. PI3K inhibitors are known to suppress spinal microglial activation by inhibiting Akt^[46]^. Activated microglia generate various mediators, including inflammatory factors and pro-inflammatory cytokines, which play critical roles in the development of chronic neuropathic pain. Inhibiting the PI3K/Akt/mTOR pathway may potentially suppress microglial activation and regulate chronic neuropathic pain^[47-49]^. Regrettably, we only assessed microglia status in the ACC after concluding the study, necessitating further research to uncover the mechanisms linking microglia status to pain hypersensitivity and emotional alterations.

The results of our current study emphasized the potential roles of PI3K-mTOR signaling pathway in the ACC in regulating pain affections and simultaneously paved the way for bridging animal study to clinical research. While this study had a few limitations. First, we relied solely on the open field test and CPA apparatus to examine exploratory behavior and mood changes. Second, cancer can cause weakening and vulnerability in rats as the disease progresses, and it is unclear to what extent this impacts the findings. Third, numerous factors can affect spontaneous activity, even though pain is a significant contributor. Lastly, it remains uncertain whether neuronal plasticity affects pain intensity and subsequently impacts emotional changes. In addition, we only observed short term about PI3K-mTOR inhibition and emotional aspects, the long-term effects were not touched. As a result, further investigations are warranted to better understand the potential mechanisms linking pain, spontaneous activity, and emotions. And to enhance the validity of the research results, different types of pain models will be added for the later research.

## Conclusions

ACC plays an important role in encoding and processing pain information, in particular pain related emotional change. PI3K-mTOR signaling is involved in this progress. Taken together, our current findings uncovered that the administration of corresponding inhibitors to the ACC can decrease protein levels of PI3K-mTOR pathway, may reduce pain intensity and subsequently improve anxiety and depressive behaviors. Our results highlight the crucial role of affective pain in regulating pain-related behaviors. Future studies focusing on optimal dosage and combinations in ameliorating pain-related negative emotion will provide a more understanding of behavioral responses and emotion alterations in cancer pain. It also provides promise for investigating potential targets or agents in modulating negative emotion.

## Data Availability

The data that support the findings of this study are available from the corresponding author, Zongming Jiang or Yuan Zhang, upon reasonable request.

## Research registration unique identifying number (UIN)

No registration for this animal study.

## Animal welfare statement

The present study followed international and our institutional guidelines for humane animal treatment and abided by relevant legislation; the present study does not involved client-owned animals, we provided humane care for all animals and decreased the number of animals used in the experiment maximally.
